# The endothelium, a key actor in organ development and hPSC-derived organoid vascularization

**DOI:** 10.1186/s12929-020-00661-y

**Published:** 2020-05-22

**Authors:** Alejandra Vargas-Valderrama, Antonietta Messina, Maria Teresa Mitjavila-Garcia, Hind Guenou

**Affiliations:** 1 INSERM UMRS-MD 1197, Université Paris Sud-Université Paris-Saclay. Hôpital Paul Brousse, Villejuif, France; 2 DHU Hépatinov, Villejuif, France; 3 UMR_S1193 Inserm. Université Paris-Saclay, Villejuif, France; 4 Université d’Evry-Val-d’Essonne. Université Paris-Saclay, Evry, France

**Keywords:** Endothelial cells, Vascularized organoids, hPSCs, Liver, Kidney, Brain, Pancreas

## Abstract

Over the last 4 decades, cell culture techniques have evolved towards the creation of in vitro multicellular entities that incorporate the three-dimensional complexity of in vivo tissues and organs. As a result, stem cells and adult progenitor cells have been used to derive self-organized 3D cell aggregates that mimic the morphological and functional traits of organs in vitro. These so-called organoids were first generated from primary animal and human tissues, then human pluripotent stem cells (hPSCs) arose as a new tool for organoid generation. Due to their self-renewal capacity and differentiation potential, hPSCs are an unlimited source of cells used for organoids. Today, hPSC-derived small intestinal, kidney, brain, liver, and pancreas organoids, among others, have been produced and are promising in vitro human models for diverse applications, including fundamental research, drug development and regenerative medicine. However, achieving in vivo-like organ complexity and maturation in vitro remains a challenge. Current hPSC-derived organoids are often limited in size and developmental state, resembling embryonic or fetal organs rather than adult organs. The use of endothelial cells to vascularize hPSC-derived organoids may represent a key to ensuring oxygen and nutrient distribution in large organoids, thus contributing to the maturation of adult-like organoids through paracrine signaling.

Here, we review the current state of the art regarding vascularized hPSC-derived organoids (vhPSC-Orgs). We analyze the progress achieved in the generation of organoids derived from the three primary germ layers (endoderm, mesoderm and ectoderm) exemplified by the pancreas, liver, kidneys and brain. Special attention will be given to the role of the endothelium in the organogenesis of the aforementioned organs, the sources of endothelial cells employed in vhPSC-Org protocols and the remaining challenges preventing the creation of ex vivo functional and vascularized organs.

## Background

In the twentieth century, the isolation and in vitro maintenance of animal and human tissues led to the establishment of most of the current cell culture techniques. In vitro two-dimensional (2D) cell culture has become widely used for fundamental cell biology research and preclinical testing. However, during recent decades, diverse studies have shown that 2D cell cultures are not fully representative of the in vivo responses of tissues and organs [1–3]. Cells growing in monolayers on a flat and rigid surface do not experience the highly complex three-dimensional (3D) environment that modulates cellular behavior and organ development through the highly complex cell-extracellular matrix (ECM) and cell-cell interactions [1, 3]. As an alternative, new cell culture techniques aiming to grow 3D cellular structures have been developed [1, 4]. At the end of the twentieth century, several publications adopted the term *organoids* to refer to 3D cell aggregates derived from animal and human cells and tissues with in vitro organ-like structures and functions [2, 5]. Despite numerous promising results [5], the shortage of human tissues and the difficulty in accessing them, together with the great heterogeneity among donors are limiting factors in the establishment of organoids from primary human cells to use as in vitro models of human organs.

The isolation of human embryonic stem cells (ESCs) in 1998 [6] and the consequent derivation of human induced pluripotent stem cells (hiPSCs) from adult somatic cells [7, 8] in 2007 revealed new virtually unlimited sources of cells for organoid generation thanks to the self-renewal capacity of these cells and their potential to differentiate into the three germ layers. Since then, the term organoid has been redefined to include the new sources of cells employed in organoid generation (human pluripotent stem cells (hPSCs), hESCs and hiPSCs, and adult stem cells) [9]. Hence, organoids can be defined today as 3D cell aggregates derived from stem cells or organ progenitors that are able to differentiate into organ-specific cell types and self-organize into structures mimicking the in vivo tissue arrangement, compartmentalization and functionality [2, 5, 10]. To date, hPSC-derived organoids (hPSC-Orgs) have been generated that resemble the small intestine [11], kidneys [12], brain [13], liver [14], pancreas [15], and others. These organoids are considered a promising model suitable for studying the mechanisms underlying human development, drug discovery, modeling diseases and regenerative medicine [9, 16, 17]. However, two main limitations remain: i) the size of the organoids is limited to a couple of millimeters due to the passive diffusion of nutrients and oxygen that threatens the survival of the cells at the organoid core, and ii) the functionality of hPSC-Orgs remains immature and resembles embryonic and fetal tissues rather than adult organs, probably due to missing developmental cues that are still unknown [13, 15, 18]. Endothelial cells (ECs) may play a key role in overcoming the aforementioned limitations.

Here, we review the progress achieved in the generation of hPSC-Orgs derived from the three primary germ layers (endoderm, mesoderm and ectoderm), which are exemplified by the pancreas, liver, kidneys and brain. We discuss the role of ECs during tissue and organ development, and we analyze the state of the art in vascularized hPSC-Orgs (vhPSC-Orgs), the source of ECs employed for this purpose and the challenges remaining in the creation of ex vivo functional and vascularized organs.

## hPSC-derived organoids and lack of maturation

Most current hPSC differentiation protocols are based on the events of in vivo embryonic development that lead to organ formation [18]. Addition of exogenous cytokines at a precise dose and time drives sequential differentiation of hPSC into a germ layer, which followed by organ-specific cell lineage commitment and cell maturation. The latter is crucial for obtaining fully functional cell types representative of in vivo human adult tissues [9]. Although there have been great advances in understanding the regulatory mechanisms underlying organogenesis, many of these are still elusive. Hence, hPSC-derived cells often have embryonic or fetal-like characteristics that are far from the much-desired mature adult cells [19–21]. The derivation of hPSC-Orgs and the consequent generation of a complex three-dimensional environment have been shown to improve the differentiation and maturation of numerous cell types [9, 15, 19, 22]. However, transcriptomic and functional analysis revealed that hPSC-Orgs are more closely related to human fetal organs [23–25], and this is true even in long-lasting cultures, such as cerebral (> 3 months of culture) [26, 27], small intestine (> 2 months) [28] or kidney organoids (> 1 month) [29].

To overcome this limitation, hPSC-Orgs of the brain [30], kidney [31], small intestine [28] or lungs [32] have been transplanted into immunodeficient mice. An increase in the size of the transplanted organoids compared to the in vitro control was observed in all these independent studies. Interestingly, brain organoids only survived in vivo when they were vascularized by the host, and their growth coincided with the beginning of organoid vascularization [30]. Perfused blood vessels of mouse origin were also found in transplanted kidney, intestine or lung organoids [28, 31, 32]. All the hPSC-Orgs showed a higher maturation in vivo*,* as evidenced by the following: functionality (e.g.*,* Ca^2+^ pattern or neuronal activity in transplanted cerebral organoids resembled early postnatal brain, while in vitro organoids represented an earlier embryonic stage), structural organization (e.g.*,* highly organized pseudostratified epithelium polarized with apical and basal surface similar to adult airways were only observed in transplanted lung organoids), increased expression of markers of maturation (e.g.*,* transplanted intestinal organoids showed an increased mRNA and protein expression markers of differentiated enterocytes, Paneth, enteroendocrine and goblet cells) and/or differentiation of cell populations not observed in the in vitro organoids (e.g.*,* fenestrated glomerular endothelium in the transplanted kidney organoids) [28, 30–32]. These results highlight the absence of all the necessary signals for proper maturation of the hPSC-Orgs in vitro. It may also be argued that in vivo organoid maturation is a consequence of longer in vivo survival (between 6 weeks and 8 months) compared to that of normal in vitro organoid cultures (between 15 and 40 days). However, prolonged cultures of similar length as transplanted organoids did not show the same maturation state than in vivo organoids of the small intestine [28], brain [30] or kidney [31] and the two latter, exhibited necrosis, expression of stress markers and reduced growth, probably due to insufficient oxygen and nutrient diffusion in vitro [13, 29–31]. Incorporation of ECs into organoids and the consequent in vitro vascularization could help organoid maturation in two ways: i) by favoring longer survival of organoid cultures thanks to the active distribution of oxygen and nutrients throughout the organoids and ii) by paracrine support of the differentiation and maturation of organ-specific cell types.

## Role of endothelial cells in the development of tissues

The cardiovascular system is the first functional organ developed during human embryogenesis beginning at approximately the third to fourth week of gestation in humans [33–35]. The formation of blood vessels and subsequent blood flow ensure the survival and development of the embryo. Far from being mere pipes supplying oxygen and nutrients to all the organs and tissues throughout the body, blood vessels are lined by a layer of mesodermal epithelial cells called endothelial cells that participate actively in several physiological processes, including organogenesis. Two nonexclusive mechanisms are responsible for tissue and organ vascularization: vasculogenesis and angiogenesis [36]. Vasculogenesis is the de novo formation of blood vessels from endothelial progenitors, the angioblasts, which converge to form a capillary plexus. This process is principally active during the first stages of embryogenesis. In contrast, angiogenesis is the process by which new blood vessels sprout from pre-existing vessels, and it is dominant in later stages of the embryonic development and in the adult stage [36]. In the kidneys, the first blood vessels sprout from the dorsal aorta and the common iliac artery as soon as the uretic bud (UB) invades the metanephric mesenchyme (MM) at E11.5 in mice. This process is followed by the association of all the artery stems to form the renal artery at E15.5 [37]. In addition, some studies suggest that in situ angioblasts may contribute to the formation of nephron capillaries that will next fuse to the kidney vasculature [38, 39]. In contrast, the central nervous system (CNS) remains avascular until the formation of the neural compartment. In mice, from E8.5 to E10, the perineural plexus (PNVP) surrounds the developing CNS without invading it. At E11.5, the periventricular plexus (PVP) branches into the subventricular zone (SVZ). The PNVP invades the neocortex at E12.5, and the new vessels fuse with the previously formed capillaries that are of PNVP origin [40, 41].

This close proximity between the endothelium and the nascent organs suggests an important role of ECs in the maturation and patterning of tissues and organs. In 2001, Lammer et al. [42] studied the role of the endothelium in the differentiation of pancreatic β-cells responsible for insulin production. An ex vivo coculture of isolated murine endoderm explants with different tissues showed that only the presence of the aortic endothelium enhanced insulin production in scattered cells. VEGFR-2 (Flk1, CD309 or KDR)^−/−^ mouse embryos, which lack blood vessels, formed a dorsal pancreas, confirmed by the presence of pancreatic progenitors (Pdx1-positive cells), but fail to grow and differentiate [43]. As a result, the dorsal pancreatic bud did not develop, the number of Pdx1-positive cells decreased over time, and no insulin nor glucagon were detected in the mutant embryos. Interestingly, the ventral pancreas showed a similar phenotype in mutant mice compare to the wildtype, suggesting ECs play an important role in the development of the dorsal but not of the ventral pancreas [43]. In addition, ectopic expression of vascular endothelial growth factor (VEGF-A) in the stomach epithelium resulted in hypervascularized areas where insulin-expressing pancreatic cells were observed [42].

The same year, Matsumoto et al. [44] detected the presence of CD31 (PECAM-1)^+^VEGFR-2^+^ ECs within the septum transversum specifically located next to the nascent hepatic cells. Using VEGFR-2^−/−^ mouse embryos and ex vivo tissue culture, the authors demonstrated that ECs not only contribute to the formation of future liver sinusoidal capillaries but also are necessary for the migration and outgrowth of the liver bud into the surrounding mesenchymal tissue. ECs have also been shown to induce hepatic specification from the endoderm as a result of the cell-cell interactions between hepatic progenitors and ECs; the latter is induced by inhibition of the Notch and WNT signaling pathways [45]. In addition, the growth of mature bile ducts alongside the vein from the core of the liver lobes towards their periphery suggests crosstalk between cholangiocyte progenitors and portal vein cells (ECs and perivascular cells) [46].

In the neocortex, the cerebral endothelium has been shown to play an active role during embryonic neurogenesis [47], favoring the differentiation of progenitors into neurons [48] and synthetizing ECM to allow neuron migration [49]. Other studies suggest that ECs may exert a niche function participating in the maintenance and self-renewal of both embryonic and adult neural stem cells (NSCs) [47], particularly at the subventricular zone (SVZ) and the subgranular zone (SGZ), which is caused by the secretion of soluble factors (e.g.*,* BDNF) [50] and ECM [51], cell-to-cell interactions [52] and the transport of stem cell-supporting substances through the bloodstream [53].

Finally, organotypic vasculature adapts to organ-specific nutrient and oxygen requirements and functionality, generating great heterogeneity along the endothelium. Together with Kupffer cells, liver sinusoidal ECs (LSECs) work as scavenger cells, taking up a variety of soluble macromolecular waste products presents in the blood (e.g.*,* fragments of collagen, hyaluronan and oxLDL), through receptor-mediated endocytosis. For this process, the liver sinusoidal capillaries have adapted with fenestrations and lack an underlying basal lamina, that is a porous endothelial barrier, which assures that solutes and colloids are delivered to the liver lobes [54]. Similarly, in the kidney, the cross talk of podocytes (i.e.*,* glomeruli epithelial cells), stromal cells and ECs generate morphologically adapted blood vessels. Thus, fenestrated capillaries without diaphragms participate in blood filtration at the glomeruli; peritubular capillaries that surround the nephron tubule and the ascending vasa recta, which are characterized by a fenestrated endothelium with diaphragms, contribute to reabsorption of water and electrolytes; and the descending vasa recta is lined by a continuous endothelium that tightly regulates the exchange of nutrients, water and gases [55]. In contrast, the ECs lining blood capillaries of the cerebral parenchyma are highly specialized cells constituting the blood brain barrier (BBB) which prevents the free passive flow of potentially harmful substances from the bloodstream to the brain. Brain or cerebral ECs (BECs or CECs) of the BBB are polarized cells characterized by complex tight junctions (TJs), principally ZO-1, claudins and occludins, membrane solute carriers and ABC-type efflux transporters. CECs are surrounded by a basement membrane, astrocyte cytoplasmic extensions and pericytes and have an estimated total surface coverage of 99%. Dysfunctional ECs appeared to contribute to the development and maintenance of pathological phenotypes such as cerebral ischemia [56] or neurological disorders [57, 58].

## Pancreas organoids and vascularization

Several different protocols for β-cell differentiation from hPSCs have been published, yet they all yield immature cells that produce low levels of insulin regardless of the chosen 2D [59, 60] or 3D [15, 61] approach [21]. In 2014, Jaramillo et al. showed that a 2D coculture of hESC-derived pancreatic progenitors (PPs) with human umbilical vein endothelial cells (HUVECs) and rat heart microvascular endothelial cells (RHMVECs) could improve PP maturation, as evidenced by higher insulin levels than what was observed in mono-cell culture or fibroblast-PP coculture. Remarkably, PP endothelial-mediated maturation was more efficient when ECs and PPs were in direct contact, suggesting a cell-to-cell interaction as the basis of β cell differentiation [62].

The first attempt to generate vascularized pancreatic organoids was reported by Takahashi et al. in 2018. Murine β cells (MIN6) [63, 64] and human isolated islets [14] formed pancreatic organoids by self-condensation together with HUVECs and mesenchymal stromal cells (MSCs). After 4 days of coculture on a Matrigel coated dish, MSC-mediated condensation of 3D self-organized aggregates was obtained. Remarkably, after being transplanted in mice, only organoids containing HUVECs developed microvasculature that was connected to the host circulatory system. Compared to the mono-culture control, the mRNA expression of multilineage human organoids was more similar to that of freshly isolated adult islets. In addition, vascularized islets outperformed nonvascularized islets in an immunodeficient mouse model of fulminant diabetes. The levels of human insulin in blood and glucose-responsive insulin secretion were higher for mice injected with coculture organoids than they were for monoculture controls.

Despite these encouraging results with primary cells, to the best of our knowledge, only one protocol for vascularizing hPSC-derived organoids has been published since then. Candiello et al. generated self-organized organoids from hPSC-derived PPs and HUVECs grown on a hydrogel. The addition of HUVECs reduced the rate of aggregation of the spheroids and their compaction. However, ECs, characterized by the expression of von Willebrand factor (vWF), did not form endothelial networks but appeared to increase the differentiation of PPs into β-cells, as evidenced by the upregulation of pancreatic islet-specific genes, PDX1 and NKX-6.1, and the β-cell insulin gene INS. The analysis of ECM proteins revealed that HUVEC-containing organoids possessed a homogeneous ECM distribution composed of Laminin, Fibronectin and Collagen IV present at both the core and the periphery of the organoids, whereas the ECM in HUVEC-devoid organoids was scarce and constrained to the outer layer of the aggregates [65].

## Liver organoids and vascularization

In 2013, Takebe et al. generated the first vascularized liver buds (LB) comprising hiPSC-derived hepatic endoderm, HUVECs and bone marrow MSCs. Following a similar approach to the one used for generating pancreatic organoids, the coculture of these three lineages on Matrigel resulted in the formation of self-organized 3D spheroids where CD31^+^ vascular networks were visible after 6 days of culture. Although these hiPSC-LBs had a fetal transcription profile, their structural configuration more closely resembled that of adult human liver than what was achieved by human hepatocytes derived from hiPSCs through other methods [66, 67]. In vivo, in a cranial window mouse model, the transplanted hiPSC-LBs underwent further maturation as confirmed by the presence of albumin positive cells (by flow cytometry) and by the detection of secreted human albumin in the serum of mice (by ELISA) 45 days after transplantation. More interestingly, 48 h after transplantation, anastomosis between human and mouse blood vessels was evident, and the resulting new vessels (12 μm in diameter) were perfused by the host [64, 67, 68]. The survival of ECs in vivo and their supportive role in hepatocyte transplantation have also been shown by other teams in a rat model of acute liver failure [32, 33] and implants of LBs under the kidney capsule of immunodeficient mice [69]. In all these studies, the control hiPSC-LB lacking ECs failed to engraft.

Other approaches attempting to vascularize LBs have been based on the use of human adipose microvascular endothelial cells (HAMECs) as a source of human ECs [70]. Recently, some studies have succeeded in generating LBs entirely from hPSCs by either a codifferentiation approach [71] or an independent differentiation of hPSCs into ECs, MSCs and hepatoblasts and their subsequent coculture [72–74]. Similar to HUVECs and HAMECs, hPSC-derived ECs have been shown to improve the maturation of hepatocytes and the engraftment of hPSC-hepatocytes in in vivo models (Ramanathan et al. 2015) [70–72, 75]. Their characterization is, in most cases, limited to the panendothelial markers VEGFR-2, CD144 (VE-Cadherin) and CD31, which hampers further comparison among the different published studies. Transcriptomic analysis of hiPSC-LBs derived by Takebe et al. revealed a heterogeneity of ECs within the resulting LBs, suggesting that ECs undergo further endothelial differentiation during coculture [24].

Recently, Goulart et al. showed that the source of ECs and MSCs used for the formation of hPSC-LBs could significantly modify the functionality of the liver organoids. Notably, they observed a higher hepatic metabolic rate in hPSC-LBs composed of hPSCs-derived ECs and dental pulp-derived MSCs (dpMSCs) compared to hPSC-LBs formed by human aortic endothelial cells (HAECs). Proteomic analysis of four different types of hPSC-LBs (HAEC/dpMSCs; HAEC/hPSC-derived MSCs; hPSC-derived ECs/dpMSCs and entirely hPSC-derived LB) revealed differences in the WNT and TGF-β pathways among the four groups. The high TGF-β expression in HAECs was related to impaired hepatocyte differentiation from hepatoblasts as shown by a decrease in hPSC-LB albumin production, which was probably caused by remodeling of the ECM [73]. In accordance with this, Koui et al. showed that hPSC-derived LSECs and hPSC-derived MSCs improved expansion, maintenance and metabolic rate of hPSC-derived hepatocytes compared to that of their primary counterparts (HUVECs and MSCs) in a 2D-coculture [74].

## Kidney organoids and vascularization

Protocols for the formation of hPSC-derived kidney organoids rapidly included hPSC-derived ECs, probably because of their shared mesoderm origin with UBs and MMs, allowing their codifferentiation. In 2014, the studies carried out by Taguchi et al. [76] combining in vivo lineage tracing in mouse embryos, ex vivo cell culture of mouse kidney progenitors and differentiation of mouse and human PSCs defined the basis for in vitro differentiation of hPSCs into kidney cell lineages and organoid formation. Their developmental approach generated embryoid bodies (EBs) and kidney hPSC-Orgs containing nephric tubules and glomeruli. Later, in 2015, a new hPSC differentiation approach based on the 2D generation of nephron progenitors that self-aggregate to form metanephric organoids was published by Morizane et al. [12]. Remarkably, although none of these studies analyzed the contribution of ECs to the final organoid phenotype, they evidenced the existence of uncharacterized cells in the interstitial space of the kidney organoids, some of which corresponded to ECs [77] as it was later confirmed by a transcriptomic study comparing these two protocols [29].

Since then, many efforts have been made to vascularize kidney hPSC-Orgs. In 2017, Freedman et al. designed a 3D hPSC differentiation protocol that yielded tubular organoids expressing nephron progenitor (NP) markers (PODXL and WT1) in capsule-like structures followed by proximal (LTL^+^) and distal (E-Cad^+^)-like tubules. Interestingly, 80% of these organoids contained CD31^+^ vWF^+^ ECs that formed cords around the tubular and capsule structures. While these organoids seemed to exhibit some of the tissue-specific markers and transport characteristics, they were limited to MM-derived lineages and did not contain collecting ducts derived from UBs. This very encouraging first approach towards the generation of hPSC-derived vascularized kidney organoids provided a path for new experimentation approaches [78]. In 2015, Takasato et al. published a new method aiming to incorporate the UB lineage that was missing in the previously described kidney organoids*.* Organoids of 6 mm in diameter were composed of approximately 500 nephrons made of glomeruli (WT1^+^), proximal (LTL^+^E-Cad^−^) and distal tubules (GATA3^−^LTL^−^E-Cad^+^) connected to a collecting duct (GATA3^+^ E-Cad^+^). The proximal tubule took up dextran in a size-selective way and showed a nephrotoxic response to a two known nephrotoxicants, cisplatin and gentamicin; however, their expression profile appeared to mimic that of fetal human kidney. These organoids also contained PDGFRα^+^ stromal cells and CD31^+^VEGFR-2 ^+^SOX17^+^ endothelial networks endowed with a lumen after 18 days of 3D culture. Remarkably, although some glomeruli contained endothelial networks, other remained avascular. In the latter group, GBM composed of laminin was observed, yet no comparison with vascularized glomeruli was performed. As in the preceding study, no further characterization of ECs or their contribution to organoid development was assessed [79, 80].

Interestingly, none of the aforementioned protocols used exogeneous VEGF-A to maintain the hPSC-derived endothelial population within the organoids, suggesting an endogenous source of this growth factor. A recent study analyzed the contribution of the different renal hPSC-derived populations in the maintenance of the organoid vasculature. Low et al. showed that VEGF-A mRNA expression and the consequent protein secretion increased throughout hPSC renal differentiation and was maintained at high levels from day 14 forward. The direct comparison between podocyte-devoid (PODXL^−^) organoids and PODXL^+^-containing organoids revealed that the latter displayed a tenfold increase in VEGF-A expression, suggesting an important role for podocytes in VEGF secretion and EC maintenance. The role of VEGF-A in the formation of organoid vasculature was further confirmed by testing VEGFR inhibitors. When an antagonist was used, the CD31^+^ networks were disrupted, while the expected development was observed in the positive controls. Variation in the total number of CD31^+^, VEGFR-2^+^, CD34^+^ ECs, however, was not evaluated. In this study, single-cell RNA sequencing and flow cytometry analyses revealed that VEGFR-2^+^-ECs present in organoids may originate from NPs (SALL1^+^SIX1^+^) and then acquire more mature endothelial characteristics, such as venous or arterial phenotypes, after extended culture. Finally, transplantation of these organoids beneath the renal capsule in NSG mice not only helped the maturation of the organoids but also triggered the formation of perfused glomerular capillaries with fenestrations formed by human and mouse ECs [39].

Recently, Homan et al. cultured d11-d14 hPSC-derived kidney organoids on a milli-fluidic device engineered with a supportive ECM to enhance the maturation of organoid vasculature. Remarkably, despite the efforts to include exogenous primary ECs such as HUVECs or human glomerular microvascular ECs, ECs succeeded in forming a vascular network within the organoids only when codifferentiated with MMs from hPSCs. In these experiments, Homan et al. showed that organoids subjected to superfusion (flow over their top surface) significantly increased the percentage of KDR^+^-endothelial progenitors and the CD31^+^ vessel percent area compared to static cultures in the absence of exogenous VEGF. EC network structures in contact with LTL^+^ tubules were increased by three-fold compared to organoids grown under static conditions. Likewise, vascular invasion of PODXL^+^ glomeruli-like compartments was enhanced in organoids under flow conditions, and capillary loops observed were similar to those found in glomeruli from E14.5 mouse kidneys. Interestingly, some of the endothelial networks were perfused through their own lumen. Together, these results suggest that microfluidics may contribute to the development and maturation of an organotypic kidney vasculature in vhPSC-Orgs [81].

## Brain organoids and vascularization

In 2013, Lancaster et al. showed that human cerebral organoids (hPSCs-COrgs) could be derived from hPSCs using a two-step differentiation process in which EB formation is followed by a neural differentiation process in Matrigel droplets maintained in a bioreactor. The expression of specific markers of the hindbrain, midbrain and forebrain highlighted that these organoids could recapitulate the morphology and partial function of the human neocortex. Despite the fact that these hPSC-COrgs were kept in culture for up to 10 months, brain development was restricted to the outer layers and extensive cell death was observed in the inner core [13], highlighting the need for vascularization to distribute nutrients and oxygen throughout the organoids.

In 2018, Mansour et al. transplanted hPSC-COrgs in a murine model and confirmed that vascularization of these organoids by the host could increase their in vivo survival [30]. Today, there exist methods to vascularize hPSC-COrgs in vitro and they can be grouped in two main strategies. First, hPSC-COrgs have been vascularized by the addition of ECs to previously formed neural progenitor cell (NPC) spheroids. Pham et al. differentiated hPSCs into ECs and added them to 34-day-old (d34)- hPSC-COrgs. An endothelial CD31^+^ network was evidenced in the outer layer of the vascularized hPSC-COrgs (vhPSC-COrgs). At day 54 of hPSC-COrg formation, hPSC-COrgs and vhPSC-COrgs were engrafted into mouse brains. Only vhPSC-COrgs survived and grew after 2 weeks of transplantation [82]. Song et al. fused hPSC-derived EC spheroids to d14 hPSC-derived NPC spheroids in the presence of primary human MSCs. Neurogenesis and spheroid regionalization were evidenced by the expression of TBR1 and MAP 2 neuron markers and GFAP^+^ glial progenitors. Inward and outward currents during voltage-clamp recording and spontaneous postsynaptic currents were detected suggesting the presence of functional neurons [83]. ECs within the vhPSCs-COrgs expressed BBB markers such as ZO-1 protein as well as GLUT1, BCRP and PGP mRNA.

In contrast, other studies showed the codifferentiation of hPSCs towards endothelial and neural lineages following EB formation [84, 85]. In the presence of exogeneous VEGF, hPSC differentiation by Ham et al. generated vhPSC-COrgs expressing markers of diverse cortical layers (e.g.*,* SOX2^+^ NSCs, DCX3^+^ or TUJ-1^+^ neurons) alongside the confirmed presence of tubular-like structures expressing ECs and CEC markers (e.g.*,* CD31, vWF, CLDN5) surrounded by α-SMA pericytes [84]. Similarly, Cakir et al. generated vascular-like CD144^+^CD31^+^vWF^+^VEGFR-2^+^ cells within brain organoids by inducing the expression of the endothelial transcription factor ETV2 at d18 of EB formation. ECs underwent further differentiation and acquired a CEC phenotype, as evidenced by TJs, which were observed by electron microscopy, immunohistochemistry (ZO-1 and OCLDN) and mRNA analysis (CLDN5 and TJP1). CEC functionality was also assessed by the expression of membrane transporters (ABCB1 and GLUT1) and a higher transendothelial electrical resistance (TEER) compared to that of hPSC-COrgs. Remarkably, although both avascular and vascularized organoids exhibited a well-organized SOX2^+^ VZ and TBR1^+^ SVZ around the lumen after 4 months in culture, vhPSC-COrgs showed higher neuronal activity, which was almost absent in hPSC-COrgs. The role of ECs in neurogenesis was further assessed by single cell RNA-sequencing analysis, revealing that vhPSC-COrg-derived neurons were more mature than those present in hPSC-COrgs. In fact, vhPSC-COrg-derived neurons showed a transcription profile similar to that of human neurons at gestational week 16–19. In vitro vascularization also seemed to be important for successful transplantation of vhPSC-COrgs in a hind-limb mouse model. The injection of dextran-FITC in the mouse bloodstream and the confocal imaging of the hPSC-COrgs and vhPSC-COrgs once explanted, confirmed that transplanted hPSC-COrgs displayed a reduced amount of perfusion although they exhibited more permeable blood vessels than the vhPSC-COrgs [85].

## Future directions

As expected from the role that ECs play during tissue and organ development in human and mouse embryos, the addition of ECs to hPSC-Orgs improved the in vitro survival of organoids and decreased the number of dead cells in the inner core. ECs also promoted the maturation of the vhPSC-Orgs, improving their metabolism and functionality by secreting soluble growth factors and ECM proteins. In vivo, the presence of human ECs enabled, in most cases, the success of the engraftment and the survival of the organoids by anastomoses between human and host vasculature. Nevertheless, the endothelial networks formed within hPSC-Orgs remained poorly characterized in most of the organoid examples reviewed herein, except for hPSC-COrgs (Table 1). The limited knowledge of organ-specific EC markers hampers the study of further endothelial differentiation and maturation, which is often reduced to the expression of panendothelial markers. Further characterization of ECs within organoids may shed some light on the mechanisms underlying EC-driven organoid maturation and EC specification.

**Table 1:** Summary of the current published protocols for vascularized hPSC-derived organoids

Organoid	ECs	Vascularization strategy	Endothelial characterization (Phenotype)	FBS	Ex. VEGF	Commentaries	Ref
Pancreas*	HUVEC	**Coculture** of HUVEC, MSCs and hPSC-derived PP progenitors. MSC-driven condensation on **Matrigel.**	• Endothelial networks • IF: CD31 • **In vivo*:*** Anastomosis of human CD31^+^ networks with mouse blood vessels	+	+	Initial % EC: 36% In vivo reperfusion and non-leaky vessels	[14, 63]
	HUVEC	**Coculture** of HUVEC and hPSC-derived PP progenitors on a hydrogel (“*amikagel”*)*.*	• IF: vWF	+	–	Initial % EC: 50% Final % EC: 45–50%	[65]
Liver**	HUVEC	**Coculture** of HUVEC, MSCs and hPSC-derived hepatic progenitors. MSC-driven condensation on **Matrigel.**	• Endothelial networks • IF: CD31 • **In vivo*:*** Anastomosis of human CD31^+^ networks with mouse blood vessels	+	+	Failure of non-vascularized (in vitro*)* organoids to engraft. Initial % EC: 11%	[67, 68]
	hPSC-EC	**Codifferentiation** of hPSC into hepatobiliary and endothelial lineage on **Matrigel.**	• Endothelial networks • IF and FACS: CD34, CD31 • IF: CD144, CD146 • mRNA expression: JAG1, NOTCH2, HES1	–	–		[71]
	HAMEC	**EB differentiation** of hepatic cells in the presence of HAMEC.	• Endothelial rosettes • IF: CD31^+^ • mRNA expression: Factor VIII, vWF and other coagulation cascade and fibrinolysis genes • **In vivo:** Human CD31^+^ in the rat spleen without migration to the liver.	+	–	Only vascularized organoids supported a sustained production of albumin in vivo up to 14 days after transplantation. Initial % EC: 30% Initial % EC: 15%	[70]
	HUVEC	**Coculture** of HUVEC, MSCs and hiPSC-derived hepatic progenitors. MSC-driven condensation on **Matrigel.**	• Endothelial networks • IF: CD31 • RNA-seq analysis • Protein analysis of the culture supernatants	+	+	Initial % EC: 45% Surface contact of HE-iPSCs with non-parenchymal cells (HUVECs and MSCs) is required for organoid morphogenesis.	[69]
	hPSC-EC HAEC	**Coculture** of HAEC or iEC, MSCs or iMSC and hiPSC-derived hepatic progenitors. MSC-driven condensation on **Matrigel.**	• Endothelial networks • FACS: CD34, CD31, CD144 at t0 • Proteomic analysis	+	+	Initial % EC: 40% Differences on the organoid metabolic rate and TGF-β and Wnt signaling pathways were observed depending on the source of EC and MSC used.	[73]
	hPSC-EC	**Coculture** of iEC, iMSCs and hiPSC-derived hepatic progenitors. iMSC-driven condensation on **Matrigel.**	• Endothelial networks • IF: CD144, CD31 • scRNA-seq analysis • **In vivo*:*** Anastomosis with host mouse blood vessels surrounded by pericytes	+	+	Completely hiPSC-derived liver organoids outperformed their counterpart made of HUVEC and MSCs. Inhibition of KDR decreased the number of endothelial networks and hampered hepatocyte maturation.	[24, 72]
**Kidney**	hPSC-EC	**Co-differentiation** of hiPSC into kidney and endothelial lineage and **self-assembling** on **transwells.**	• Endothelial networks with lumen • IF: KDR, CD31, SOX17	–	–		[79, 80]
	hPSC-EC HUVEC	**Co-differentiation** of hiPSC into kidney and endothelial lineage. **Self-assembling** in low-attachment plates and further culture on **transwells.**	• Endothelial networks with lumen • IF: KDR, CD31, CD34 • scRNA-seq analysis: Different subsets of EC • **In vivo*:*** Perfused and fenestrated vessels	+	–	Maximum 3% of EC at d14	[39]
	hPSC-EC	**Co-differentiation** of human pluripotent epiblast spheroids into kidney and endothelial lineage on **Matrigel/Collagen gels.**	• Endothelial cords around capsule and tubular structures • IF: CD31, vWF	–	–	Organoids survived for up to 2 months.	[78]
	hPSC-EC, HUVECS, HNDFs, and adult GMECs	**Co-differentiation** of hiPSC into MM and endothelial lineage in suspension. From d11 to d14, organoids were culture on **perfused chips.**	• Endothelial networks with lumen • IF and FACS: CD31, MCAM and KDR • mRNA expression: CD31	+	–		[81]
**Brain**	hPSC-EC	**EB co-differentiation** of neural progenitors and endothelial cells.	• Vascular-like structures at d30; • IF: CD144, CD31, vWF, KDR and tight junctions^:^ OCLN, αZO-1 • EM: Tight junctions • mRNA expression: CD144, CD31, KDR, TEK, vWF, OCLN, CD34, CLDN5, OCLN, TJP1, ABCB1 and GLUT1 • In vitro perfusion with Dextran-FITC. • TEER: 351 ± 10 Ω cm^− 2^ • scRNA-seq analysis • **In vivo*:*** Anastomosis of human CD31^+^ networks with mouse blood vessels	+	–	EC came from modified hiPSC expressing ETV2 from day 18 of differentiation. Organoids survived up to 4 months and reached 3,5–4 mm of diameter. Vascularized organoids displayed better neural differentiation and neural functions.	[85]
	hPSC-EC	**EB co-differentiation** of neural progenitors and endothelial cells in low-attachment plates and further, embedded in **Matrigel droplets**.	• Tubule-like structures • IF: CD31, vWF, CLDN5 • mRNA expression: ANGPT1, CD31 • RNA-seq analysis: 106 TJ-related genes and GPR124	+	+	Organoids survived up to 4 months. α-SMA pericytes surrounded CD31^+^ EC.	[84]
	hPSC-EC	**Fusion of** hPSC-derived cortical and vascular **spheroids**.	• IF: CD31, CD144, Z0–1 • FACS: CD31 • mRNA expression: BCRP, PGP, GLUT-1	–	–	NPSC: EC: hMSC (1:2:3) Organoids survived up to 2 months.	[83]
	hPSC-EC	**Co-culture** of d34-brain organoids with ECs in **Matrigel.**	• Tubular structures • IF: CD31 • **In vivo*:*** human CD31^+^ vessels.	+	+	Organoids survived up to 2 months. Only in vitro vascularized organoids survived in vivo 2 weeks after transplantation.	[82]

Organ, endothelial cell source, vascularization strategy, endothelial characterization, use of fetal bovine serum (FBS) and exogenous VEGF (Ex. VEGF) are described for every vhPSC-Orgs protocol. IF: Immunofluorescence. FACS: fluorescence activated cell sorting. scRNA-seq analysis: single cell RNA sequencing analysis. *For pancreas organoids, authors often refer to pancreatic islet organoids because of the missing exocrine compartment. ** Liver organoids are also known in most cases as liver buds (LB)

The generation of fully hPSC-derived organoids may be advantageous for the creation of in vitro human organ models with a homogeneous genetic background, and opens the possibility of using organoid autografts in regenerative medicine; however, currently, many vascularized organoids are made of primary ECs (e.g.*,* HUVEC or HAECs) partly because of their great potential for in vitro proliferation, allowing the generation of large quantities of cells. Despite this, the use of EC derived from hPSCs have been proven to be more suitable for organoid maturation. Two independent studies showed that different sources of ECs can result in different hepatocyte maturation [73, 74], with hPSC-derived ECs being the most advantageous source. This was partially explained by the fact that hPSC-derived ECs used in these studies were differentiated into liver-specific ECs, LSEC, from hPSCs prior to organoid formation [74]. Other studies suggested that hPSC-derived ECs may have an immature endothelial progenitor-like phenotype, and thus, the addition of exogenous molecules or the coculture with organ-specific cells could induce further organotypic vascular specification [74, 86–88].

**Fig. 1:**
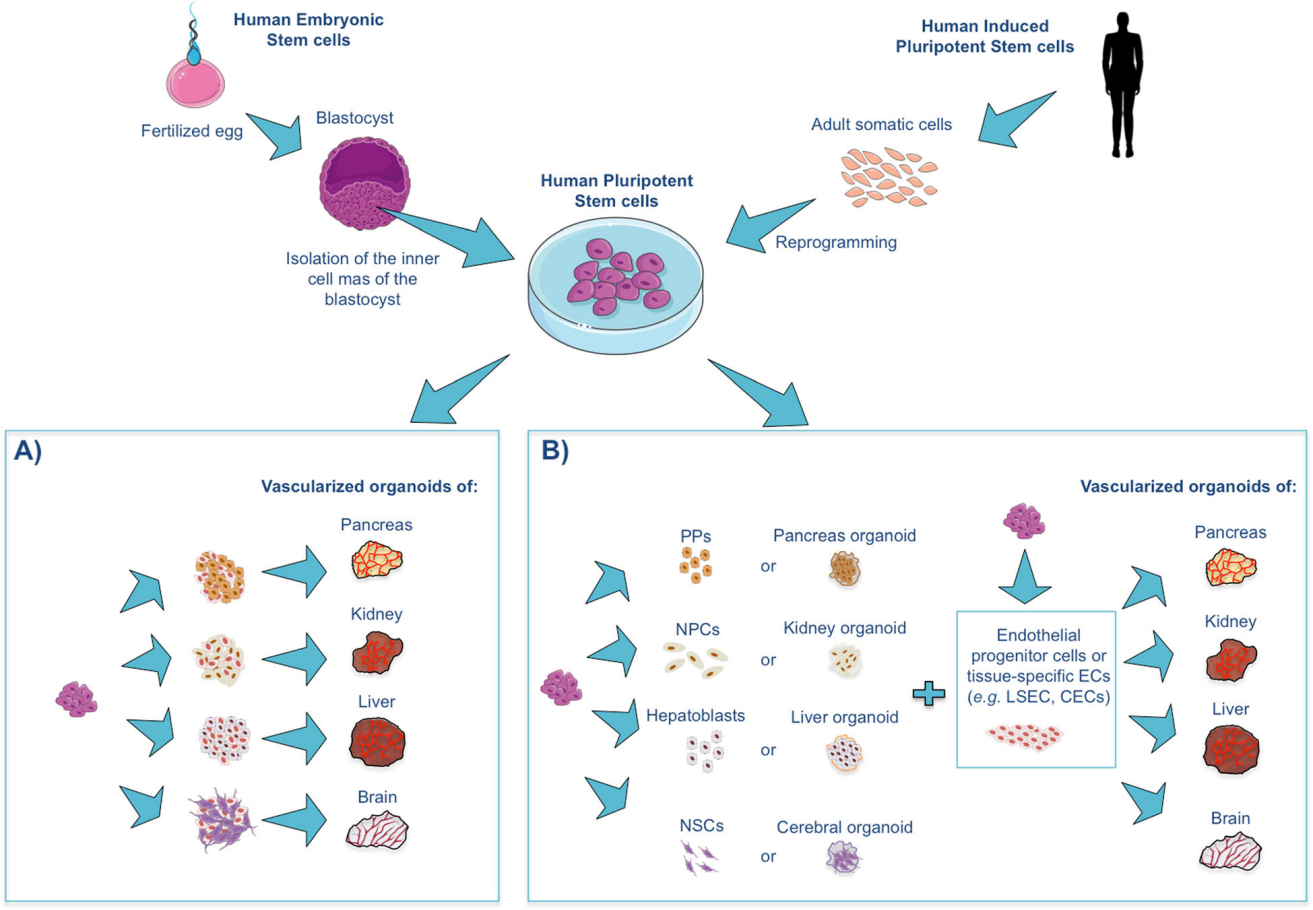
Strategies for the vascularization of hPSC-derived organoids. hPSC-derived endothelial cells (ECs) present in the vascularized organoids can be obtained by **a)** codifferentiation of human pluripotent stem cells (hPSCs) into all the cell lineages forming the organoid or **b)** independent differentiation of hPSCs into ECs and tissue progenitors for further coculture. hPSC-derived ECs can also be added to preformed organoids. PPs: pancreas progenitors; NP: nephron progenitors; NSCs: neural stem cells; LSECs: liver sinusoidal endothelial cells; and CECS: cerebral endothelial cells

Efforts made in vascularizing hPSC-Orgs with hPSC-ECs have followed two main approaches which are summarized in Fig. 1. In one approach, hPSCs are independently differentiated towards either endothelial progenitors or tissue-specific ECs (e.g.*,* LSECs or CECs) and tissue-specific cell types (e.g.*,* hepatocytes, NPs or UBs, NPCs). Their further coculture allows the control of the initial cell type ratio in each organoid and the incorporation of scaffolds. This approach, however, raises the question about timing of coculture: should it be performed with tissue progenitors or should ECs be added once the tissue organoids are established? In the other approach, hPSCs are codifferentiated towards all organ-specific cell types, including ECs, which typically follows EB formation. In this approach, organoid self-organization aims to mimic human embryonic development, but the percentage of ECs and the organoid architecture can hardly be controlled. In addition, this methodology is not compatible with differentiation protocols where inhibitors of mesoderm differentiation (e.g. NOGGIN) are required (Fig. 1).

It is worth mentioning that although we have described hPSC-Orgs containing EC networks, none of them has been perfused in vitro and therefore, cannot be considered completely vascularized. Recently, an American research team engineered perfused in vitro high cellular density tissues derived from avascular hPSC-EBs, cerebral organoids and cardiac spheroids embedded in ECM*.* Using a 3D printing method (sacrificial writing), they printed gelatin into organoid or EB-derived functional tissues at 4 °C. At 37 °C, the gelatin melted leaving behind tubular channels that could be perfused, in between highly packed EBs or organoids whose morphology was not affected. This method ensured the in vitro survival of large tissues (> 2.5 ml in volume and ≈4 mm in thickness) with maintained functionality, but they lacked ECs [89]. The authors claimed that the generated vascularized cardiac tissues had higher contractility, enhanced beating capability and improved beating synchronization. These tissues also responded to perfused molecules known to increase the beating frequency or reduce the contractile amplitude. These results, together with the fact that in vivo perfusion has been related to successful organoid engraftment, survival and maturation, suggest that technologies making possible in vitro perfusion of hPSC-Orgs (e.g. tissue-engineered blood vessels, scaffolds or bioreactors) may contribute to the production of large vhPSC-Orgs [81, 90, 91]. Additional fine-tuning of shear stress and use of mural cells may help with generating and stabilizing specific organotypic vasculature within vhPSC-Orgs.

Finally, the production of vhPSC-Orgs with a size suitable for therapeutic use in humans depends on the development of cost-efficient technologies. The maintenance of hPSCs and their differentiation into the desired vhPSC-Orgs often requires the use of expensive growth factors, cytokines and proteins. In recent decades, encouraging cytokine-free protocols for hPSC differentiation to some lineages have been achieved using small molecules [92, 93]. In this review, we also showed some examples in which the ECs and stromal cells of vhPSC-Orgs synthesized their own ECM, which could replace use of exogenous ECM proteins in the future [65, 79, 85]. New methods to grow and differentiate hPSCs at a large scale in bioreactors are now also possible [94]. Hence, the creation of cost-efficient and large-scale vhPSC-Orgs seems to be a real possibility in the near future.

## Conclusion

In conclusion, the studies reviewed herein suggest an encouraging future for the generation of vascularized hPSC organoids not only for the aforementioned organs but also for the intestine, heart, skin or lungs. Further investigations are necessary to determine organ-specific markers of ECs that may be used in the identification of an organotypic vasculature differentiated within the vhPSC-Orgs. Tissue engineering technologies, such as scaffolds, bioprinting or organoids-on-a-chip devices, could help to the development of these physiological and pathological models relevant for fundamental and clinical research, regenerative medicine and pharmacology.

## Data Availability

Not applicable.

## Abbreviations

2D: Two-dimensional
3D: Three-dimensional
BBB: Blood brain barrier
BECs: Brain endothelial cells
BMP: Bone morphogenetic protein
CECs: Cerebral endothelial cells
CKD: Chronic kidney disease
CNS: Central nervous system
dpMSCs: Dental pulp-derived MSCs
E: Embryonic day
EB: Embryoid body
ECM: Extracellular matrix
ECs: Endothelial cells
GBM: Glomerular basement membrane
HAEC: Human aortic endothelial cells
HAMEC: Human adipose microvascular endothelial cells
hESCs: Human embryonic stem cells
hiPSCs: Human induced pluripotent stem cells
hPSC-COrgs: hPSC-derived cerebral organoids
hPSC-Orgs: hPSC-derived organoids
hPSCs: Human pluripotent stem cells
HUVEC: Human umbilical vein endothelial cells
LB: Liver buds
LSECs: Liver sinusoidal endothelial cells
MM: Metanephric mesenchyme
MSCs: Mesenchymal stromal cells
NP: Nephron progenitors
NPCs: Neural progenitor cells
NSCs: Neural stem cells
PNVP: Perineural plexus
PP: Pancreatic progenitors
PVP: Periventricular plexus
RHMVECs: Rat heart microvascular endothelial cells
SGZ: Subgranular zone
SVZ: Subventricular zone
TEER: Transendothelial electrical resistance
TJs: Tight junctions
UB: Uretic bud
VEGF-A: Vascular endothelial growth factor A
VEGFR-2: Vascular endothelial growth factor receptor-2
vhPSC-COrgs: Vascularized hPSCs-derived cerebral organoids
vhPSCs-Orgs: Vascularized hPSC-derived organoids
vWF: von Willebrand factor
